# The Cinderellas of the scanner: Magnetic resonance imaging ‘pre-scan’ and ‘post-scan’ times: Their determinants and impact on patient throughput

**DOI:** 10.4102/sajr.v24i1.1946

**Published:** 2020-12-01

**Authors:** Marthinus B. van Rooyen, Richard D. Pitcher

**Affiliations:** 1 Division of Radiodiagnosis, Department of Medical Imaging and Clinical Oncology, Faculty of Medicine and Health Sciences, Stellenbosch University, Cape Town, South Africa; 2 Division of Radiodiagnosis, Department of Medical Imaging and Clinical Oncology, Faculty of Medicine and Health, Stellenbosch University, Cape Town, South Africa

**Keywords:** imaging workflow, magnetic resonance imaging, determinants, service efficiency, ANOVA

## Abstract

**Background:**

Increasing demand for magnetic resonance imaging (MRI) has contributed to extended patient waiting times worldwide. This is particularly true in resource-limited environments, prompting this institutional workflow analysis.

**Objective:**

To determine the ‘pre-’ and ‘post-scan’ times for normal-hour MRI studies conducted at a tertiary-level, public-sector South African hospital and to assess any association with demographic details, patient characteristics, anatomical site and scan parameters. A secondary objective was determination of the average daily MR ‘down’ time.

**Methods:**

A prospective descriptive study stratifying MRI workflow into ‘pre-scan’, ‘scan’, ‘post-scan’ and ‘down’ times. During ‘pre-‘ and ‘post-scan’ times patients occupied the scanner whilst staff performed tasks indirectly contributing to image acquisition. During ‘down’ time no patient occupied the MRI room. ‘Pre-’ and ‘post-scan’ times were compared with demographic details, patient characteristics, anatomical site and study parameters, utilising correlation analysis or analysis of variance (ANOVA).

**Results:**

A total of 223 patients (*n* = 223) underwent 286 investigations in the 23-day review period. Seventy per cent of routine working time was utilised in image acquisition. The ‘pre-’ and ‘post-scan’ times together accounted for 19% and ‘down’ time for 11% of working time. Prolonged ‘pre-’ and ‘post-scan’ times were independently associated with age less than 12 years, anaesthesia, sedation and immobility (*p* < 0.01 in all cases). The longest median combined ‘pre-’ and ‘post-scan’ time by anatomical site (cholangiopancreatography, 21:46 min) was more than six times the shortest (pituitary fossa, 3:11 min).

**Conclusion:**

A critical analysis of magnetic resonance ‘pre-’ and ‘post-scan’ times can provide valuable insights into opportunities for enhanced service efficiency.

## Introduction

There is an increasing global demand for radiological services, including magnetic resonance imaging (MRI).^1,2,3,4,5^ Continual technological advances, ongoing evolution of imaging sequences and the absence of ionising radiation contribute to ever-increasing clinical applications for MRI. Recent advances in breast, prostate, cardiac and hepatobiliary imaging have contributed to a relentless increase in service requests.^1,6,7^

Burgeoning clinical demand in the face of finite equipment resources has resulted in extended MRI waiting times worldwide. This is especially true in the public healthcare services of low- and middle-income countries (LMICs),^5,6^ including South Africa (SA). On an average, countries of the Organization for Economic Co-operation and Development (OECD) have 70-fold more MRI units per million people than the SA public healthcare sector. Furthermore, the SA private sector has 46-times the MRI resources of the country’s public sector.^1^ Service pressures in the SA public sector are compounded by the quadruple disease burden of trauma, infection (tuberculosis, human immunodeficiency virus), non-communicable diseases and maternal- and child disorders.^8,9,10^

These clinical demands are exemplified at Tygerberg Hospital (TBH), the 1386-bed, tertiary-level teaching hospital of the Faculty of Medicine and Health Sciences of Stellenbosch University, in Cape Town, SA. The TBH MRI service was commissioned in 2002, with a single 1.5 Tesla machine. This was replaced a decade later by a new unit with the same magnet strength. Throughout the 18 years of the service, there have been sustained efforts to enhance efficiency, extend operating hours and increase patient throughput.^6^ These have been successful, to the extent that the annual caseload has increased 147% on a single machine with the same staff complement. Nonetheless, the relentless increase in clinical demand has ensured progressive lengthening of the outpatient waiting time, which reached 128 working days or almost 6 months, at the commencement of the study. The MRI workflow was thus critically evaluated, with a view to enhancing efficiencies wherever possible.

Since radiological equipment only fulfils its healthcare role when generating diagnostic images,^11,12^ time not directly devoted to image acquisition should be kept to a minimum.^13^ Optimising patient throughput, defined as the cycling of patients through a hospital’s fixed resources, is an essential operations management strategy.^14^ To assess the efficiency of MR patient throughput, we adopted a notional sequence of simple workflow steps relative to the period of image acquisition.

The ‘pre-scan’ interval is from patient entry into the scanning room to commencement of the scan; the ‘scan’ time is from start to end of image acquisition; the ‘post-scan’ period is from scan completion to patient departure from the scanner; ‘down’ time is when no patient occupies the MRI room.

Scan time is a function of the imaging sequences used. These are dictated by the diagnostic protocol required, which is informed by the clinical question. The time required for any imaging sequence or protocol is usually machine-specific and cannot be reduced without compromising diagnostic detail. It is in this context that a critical evaluation of ‘pre-’ and ‘post-scan’ times assume particular significance when looking to enhance patient throughput.

Despite increasing MR service pressures worldwide, there has been little work on the determinants of MR ‘pre-’ and ‘post-scan’ times. This is surprising, since such knowledge is imperative for assessment of MRI operational efficiency.^3,7,8,11,12,13,14,15,16^

The primary aim of this study was therefore to determine the ‘pre-’ and ‘post-scan’ times for normal-hour MRI studies conducted at a tertiary-level, public-sector SA hospital and to assess any association with demographic details, patient characteristics, anatomical site and scan parameters. A secondary aim was determination of the average daily MR ‘down’ time.

## Materials and methods

This prospective study was conducted at TBH from 4 June through 4 July 2019. All MRI studies performed during normal weekday working hours (Monday–Friday: 07h30–16h00) were included. Data were recorded on a customised data sheet, including patient age and gender, referring clinician, examination urgency, patient mobility, anaesthesia/sedation, MRI examination(s), contrast administration and the timestamps for the four sequential MR workflow steps. The TBH MRI unit is a 1.5 Tesla Siemens Magnetom Aera, (Siemens Healthineers, Germany).

Immobile patients included those requiring precautions for suspected spinal cord injuries, those with decreased level of consciousness and frail patients. ‘Pre-scan’ actions included patient transfer to, and positioning on the MRI bed, as well as coil placement, protocol selection and sedation or anaesthetic induction, as required. ‘Post-scan’ activities were coil removal, reversal of anaesthesia, and patient assistance from the bed to the scanning room door. The ‘pre-’ and ‘post-scan’ times therefore included all tasks contributing indirectly to image acquisition. ‘Down’ time was the interval between exit of the preceding and entry of the succeeding patient, during which no patient was in the scanner. All time intervals were recorded in minutes and seconds.

A research assistant recorded all time stamps for each patient. For quality assurance, the start and end of image acquisition for each patient were cross-referenced on the institutional picture archiving and communication system (PACS), the Phillips IntelliSpace PACS Enterprise 4.4, Phillips Medical Systems, The Netherlands. Anaesthetic staff, porters and nurses were unaware of the study.

Summary statistics of the ‘pre-’ and ‘post-scan’ times were reported with medians and interquartile ranges. For categorical variables, frequency counts and percentages were reported. Comparisons of scan times between different patient groupings were done using one-way analysis of variance (ANOVA). The times were found to deviate from the assumption of normality, and all ANOVA analyses were therefore done on Box-Cox transformed scan times. Where necessary, two-way ANOVAs were performed for more insight into reasons for observed time differentials. Studies performed less than three times in the review period were excluded from statistical analysis. Statistical version 13 (TIBCO Software Inc. 2018) was utilised for analysis.

### Ethical consideration

The study was approved by the Health Research Ethics Committee of Stellenbosch University (HREC number S19/01/017). Approval was with waiver of informed consent, since study data were confined to workflow analysis and included no patient details. Patient anonymity was assured through the use of a unique study identifier known only to the principal investigator. Clinical management was not impacted in any way.

## Results

### Patient profile

A total of 223 patients (*n* = 223; females = 113, 51%) with a median age 38 years [interquartile range (IQR): 16–56 years] were scanned in 23 working days, at an average of 9.7 patients per day.

More than three-quarters of the cohort (*n* = 175, 79%) had one investigation, whilst almost one-fifth (*n* = 39; 18%) had two studies and less than 5% (*n* = 9, 4%) underwent more than two investigations.

Intravenous contrast was administered to almost half (*n* = 93, 42%), and approximately one-quarter (*n* = 52, 23%) required either anaesthesia (*n* = 20; 9%) or sedation (*n* = 32, 14%). More than one-fifth (*n* = 48, 22%) had limited mobility; forty-eight patients were less than 12 years of age (22%).

More than 90% of the anaesthetised group (*n* = 19/20, 95%) and more than 80% of sedated patients (*n* = 27/32, 84%) were younger than 12 years. Emergencies comprised just over 10% (*n* = 24, 11%).

### Study profile

A total of 286 (*n* = 286) investigations were performed, including 31 different studies, at an average of 12.4 examinations per day. Three studies, namely MR brain (*n* = 69, 24%), cervical spine (*n* = 53, 19%) and lumbar spine (*n* = 46, 16%), together accounted for almost 60% of all investigations (168, 59%). The paediatric (*n* = 48/223, 22%), neurosurgical (*n* = 26/223, 12%), orthopaedic (*n* = 24/223, 11%) and neurology departments (16/223, 7%) were responsible for more than half the referrals (114/223, 51%).

### Overall time utilisation

Of the 11730 working minutes in the review period, approximately 70% (8178 min) was utilised for image acquisition, equating to a daily average of 356 min or just less than 6 h. The mean image acquisition time was 29 min per examination.

The ‘pre-scan’ and ‘post-scan’ times together constituted almost one-fifth of available working time (2259 min; 19%) averaging 98 min daily, with ‘pre-scan’ time (1595 min) more than double ‘post-scan’ time (664 min).

Downtime accounted for a little more than 10% (1293 min, 11%) of available time, averaging almost 1 h (56 min) daily. The median ‘pre-scan’ and ‘post-scan’ times (IQR) were 4:08 min (2:39, 8:26) and 2:03 min (1:30, 3:28), respectively. The data are presented in Table 1.

**TABLE 1:** Pre-scan and post-scan times for different patient and magnetic resonance imaging scan variables.

Examination	Outcome	*n*	%	Pre-scan time			Post-scan time			Combined pre-scan and post-scan time		
				Median	IQR (min:s)	*p*	Median	IQR (min:s)	*p*	Median	IQR (min:s)	*p*
All scans	-	223	-	4:08	2:39–8:26	-	2:23	1:30–3.28	-	6:52	4:38–13:03	-
One MRI	-	-	-	-	-	-	-	-	-	-	-	0.08
	Y	175	78	4:38	2:44–9:59	0.04	2:24	1:30–3:28	1	7:15	4:59–13:30	-
	*N*	48	22	3:16	2:15–5:13	-	2:21	1:28–3:27	-	5:41	4:11–8:59	-
Age	-	-	-	-	-	-	-	-	-	-	-	< 0.01
	< 12	48	21	13:10	10:31–19:16	< 0.01	2:49	1:49–4:53	< 0.01	17:16	12:45–23:51	-
	> 12	175	79	3:25	2:19–5:15	-	2:16	1:28–3:15	-	5:45	4:08–8:29	-
Immobile	-	-	-	-	-	-	-	-	-	-	-	< 0.01
	Y	48	22	4:42	3:21–10:55	0.1	2:56	1:54–4:42	0.02	9:26	5:35–15:17	-
	*N*	175	78	4:04	2:31–7:22	-	2:12	1:29–3:12	-	6:23	4:15–12:50	-
Anaesthetic	-	-	-	-	-	-	-	-	-	-	-	< 0.01
	Y	20	9	17:58	11:32–22:37	< 0.01	4:22	1:46–6:21	< 0.01	21:34	15:15–28:36	-
	*N*	203	91	3:46	2:32–6:20	-	2:19	1:29–3:16	-	6:20	4:18–10:40	-
Sedation	-	-	-	-	-	-	-	-	-	-	-	< 0.01
	Y	32	14	13:53	10:52–18:14	< 0.01	2:30	1:50–4:02	0.06	17:38	12:59–22:02	-
	*N*	191	86	3:38	2:30–5:46	-	2:19	1:29–3:26	-	6:11	4:15–9:46	-
Emergency	-	-	-	-	-	-	-	-	-	-	-	0.73
	Y	24	11	3:37	2:19–6:25	0.29	2:50	1:28–4:37	0.43	6:20	5:03–9:49	-
	*N*	199	89	4:10	2:43–9:26	-	2:20	1:30–3:24	-	6:58	4:25–13:30	-
Contrast	-	-	-	-	-	-	-	-	-	-	-	0.63
	Y	93	42	4:53	2:31–11:35	0.26	2:17	1:24–3:38	0.52	7:56	4:08–14:02	-
	*N*	130	58	4:01	2:42–6:01	-	2:24	1:32–3:26	-	6:47	5:00–11:32	-
One MRI: Brain	-	-	-	-	-	-	-	-	-	-	-	0.05
	Y	50	28	6:01	2:59–11:51	0.05	2:28	1:29–3:43	0.26	11:36	5:25–17:35	-
	*N*	125	72	4:09	2:40–6:55	-	2:22	1:32–3:19	-	6:50	4:15–11:40	-
One MRI: Cervical spine	-	-	-	-	-	-	-	-	-	-	-	0.04
	Y	18	10	3:00	1:59–3:55	< 0.01	1:59	1:29–4:18	0.64	5:22	4:13–7:20	-
	*N*	157	90	5:06	2:55–11:00	-	2:25	1:32–3:26	-	7:35	5:07–13:51	-
One MRI: Lumbar spine	-	-	-	-	-	-	-	-	-	-	-	0.07
	Y	13	7	2:08	2:02–3:56	< 0.01	2:28	0:46–3:10	0.08	4:08	2:12–7:02	-
	*N*	162	93	4:52	2:59–10:34	-	2:25	1:35–3:29	-	7:26	5:07–13:51	-
Whole spine	-	-	-	-	-	-	-	-	-	-	-	0.09
	Y	5	3	4:57	4:42–6:25	0.55	5:50	3:28–12:50	< 0.01	19:16	8:10–21:58	-
	*N*	175	97	4:38	2:44–9:59	-	2:24	1:30–3:28	-	7:15	4:59–13:30	-
One MRI: Cardiac	-	-	-	-	-	-	-	-	-	-	-	0.93
	Y	8	5	6:12	4:56–7:34	0.4	3:42	2:15–5:12	0.27	10:29	6:53–13:30	-
	*N*	167	95	4:23	2:41–10:08	-	2:24	1:30–3:22	-	7:10	4:50–13:51	-
One MRI: IAM	-	-	-	-	-	-	-	-	-	-	-	0.03
	Y	6	4	1:53	1:38–2:31	< 0.01	1:26	1:16–1:33	0.01	3:34	3:06–3:55	-
	*N*	169	96	4:47	2:53–10:09	-	2:25	1:37–3:30	-	7:25	5:10–13:41	-
One MRI: Knee	-	-	-	-	-	-	-	-	-	-	-	0.69
	Y	5	3	5:16	4:42–5:19	0.73	2:28	2:24–2:31	0.65	7:06	6:48–7:44	-
	*N*	170	97	4:34	2:42–10:09	-	2:22	1:30–3:28	-	7:17	4:53–13:32	-
One MRI: Prostate	-	-	-	-	-	-	-	-	-	-	-	0.06
	Y	5	3	13:15	11:56–18:05	< 0.01	2:25	2:12–3:12	0.87	15:08	14:23–21:32	-
	*N*	170	97	4:33	2:42–8:35	-	2:24	1:30–3:28	-	7:07	4:53–13:06	-
One MRI: Rectum	-	-	-	-	-	-	-	-	-	-	-	0.88
	Y	4	2	4:23	4:10–5:34	0.97	2:31	2:07–2:44	0.95	7:02	6:17–8:17	-
	*N*	171	98	4:41	2:43–10:08	-	2:24	1:30–3:29	-	7:15	4:56–13:35	-
One MRI: Cervix	-	-	-	-	-	-	-	-	-	-	-	0.66
	Y	4	2	3:31	3:11–4:41	0.57	2:53	1:47–3:58	0.72	6:15	5:19–8:19	-
	*N*	171	98	4:41	2:43–10:08	-	2:24	1:30–3:24	-	7:20	4:57–13:35	-
One MRI: Neck	-	-	-	-	-	-	-	-	-	-	-	0.15
	Y	4	2	10:23	2:43–9:26	0.24	6:16	2:59–8:05	0.11	17:14	8:29–26:12	-
	*N*	171	98	4:38	2:42–9:26	-	2:24	1:30–3:22	-	7:14	4:59–13:28	-
One MRI: Pituitary Fossa	-	-	-	-	-	-	-	-	-	-	-	< 0.01
	Y	4	2	2:08	1:19–2:32	< 0.01	1:17	00:41–1:58	0.03	3:11	2:44–3:44	-
	*N*	171	98	4:42	2:52–10:08	-	2:25	1:33–3:28	-	7:22	5:04–13:35	-
One MRI: Shoulder	-	-	-	-	-	-	-	-	-	-	-	0.71
	Y	4	2	5:14	4:31–9:44	0.59	2:16	1:41–4:27	0.83	6:56	6:20–14:02	-
	*N*	171	98	4:35	2:43–9:59	-	2:16	1:30–3:28	-	7:15	4:57–13:30	-
MRCP	-	-	-	-	-	-	-	-	-	-	-	0.03
	Y	3	2	17:38	8:35–23:00	0.03	5:01	4:08–5:27	0.08	21:46	14:02–28:01	-
	*N*	172	98	4:34	2:42–9:02	-	2:22	1:30–3:22	-	7:10	4:57–13:09	-

Note: Analysis of variance (ANOVA) and Box-Cox transformed scan times. *P* value (*p* < 0.05) were statistically significant.

MRI, magnetic resonance imaging; IQR, interquartile range; MRCP, magnetic resonance cholangiopancreatography; min, minutes; s, seconds; Y, yes; N, no.

Patients less than 12 years of age, those requiring anaesthesia or sedation and those with decreased mobility were associated with *increased* ‘pre-’ and ‘post-scan’ times, whilst studies of the internal auditory meatus and pituitary fossa were associated with significant shortening of both periods.

Investigations with significant prolongation of ‘pre-scan’ time alone were MR cholangiopancreatography (MRCP) and MR prostate, whilst studies of the cervical and lumbar spine were associated with significantly shortened pre-scan times. More than one investigation and intravenous contrast administration, did not impact the ‘pre-’ or ‘post-scan’ times.

The longest median combined ‘pre-’ and ‘post-scan’ time by anatomical site (MRCP, 21:46 min) was more than six times that of the shortest (pituitary fossa, 3:11 min), whilst the longest median combined ‘pre-’ and ‘post-scan’ time by scan parameter (anaesthetised patients, 21:34 min) was more than three times that of non-anaesthetised patients.

## Discussion

To our knowledge, this study presents a unique stratification of MR workflow. The key finding is that activities not directly contributing to image acquisition accounted for approximately 30% of available working time. Such knowledge provides important insights into opportunities for improved efficiency and is particularly pertinent in resource-constrained environments.

The study also provides the first detailed assessment of the impact of patient profile, study type and ancillary scan parameters on patient throughput. Importantly, the median ‘pre-scan’ time and combined ‘pre-’ and ‘post-scan’ times for studies of different anatomic sites can vary by a factor of more than six.

The identification of studies with significantly prolonged ‘pre-scan’ or combined ‘pre-’ and ‘post-scan’ times allow detailed interrogation of the workflow, with a view to intervention. For example, many factors could be implicated in the prolonged ‘pre-scan’ time of the prostate examination. Firstly, this requires fairly detailed patient explanation and reassurance. Additionally, pre-procedure intravenous spasmolytics are utilised, in conjunction with a rectal catheter, for elimination of rectal gas. Furthermore, MR prostate and MRCP in our analysis both require intravenous contrast administration by electric pump, which in turn involves careful preparation and calibration. Similarly, the MRCP protocol is characterised by short MRI sequences (less than 96 s), which are highly susceptible to breathing motion artefact. Breath-holding techniques must therefore be explained and rehearsed until full patient compliance is achieved.

Additionally, MR services involving a significant number of children or immobile patients, and those including anaesthetic/sedation lists can expect decreased throughput relative to services with a predominantly ambulant adult population. These are important considerations when evaluating equitable access to limited public-sector resources, particularly for the paediatric population.

Our finding that ‘pre-scan’ time is independent of the number of examinations performed on a single patient appears at first glance to be counter intuitive. However, it is a key finding. We have highlighted that ‘pre-scan’ time is not a factor of the *number* of examinations, but rather related to other considerations such as the *nature* of the MR study and patient characteristics.

Notwithstanding the unique MR workflow steps defined in this study, the analysis was broadly aligned with ‘lean management’ principles. The ‘lean’ approach was developed in the automotive industry and systemised in the Toyota Production System of the 1930s.^5,15^ Since then, it has been applied in many sectors, including healthcare.^5,15,16^ It strives to enhance efficiency and productivity through the elimination of all forms of workflow ‘waste’ and the optimisation of steps that add value.

From a ‘lean management’ perspective, only ‘scan’ time is perceived to add value, whilst ‘pre-’ and ‘post-scan’ activities are construed as workflow ‘waste’. ‘Lean’ principles dictate that ‘pre-’ and ‘post-scan’ activities be completed outside the scanner, wherever possible. This is a crucial notion when looking to optimise MR efficiency. Newer MRI units are now available with a stationary magnet and detachable table. This creates the opportunity for complete patient preparation outside the scanner room, with subsequent transfer to the scanning room on the detachable table, for docking onto the stationary magnet. Our findings suggest that the capital investment in an MR unit that includes duplicate coils, an anaesthetic machine and a detachable table, together with suite design that incorporates separate ‘preparation’ and ‘recovery’ rooms could enhance throughput by approximately 40%. Such a configuration would minimise ‘pre-’ and ‘post-scan’ time and potentially eliminate ‘downtime’. Comprehensive workflow changes of this nature are difficult to implement in an established service, with no prospect of a new unit with detachable table or redesign of the floor plan. Nonetheless, lean management involves sustained commitment to iterative shortening of all activities contributing to the ‘pre-’ and ‘post-scan’ times for each examination.

Our study suggests that current downtime represents a further opportunity for workflow optimisation. We identified an average of 56 such minutes daily. Elimination would augment scanning time by approximately 242 h annually, facilitating a further 500 scans per year and potentially significantly reduce the current outpatient waiting time of 128 days. Conversely, it could be argued that *some* MR ‘downtime’ is acceptable, and even necessary, in the normal MR working day, facilitating minor administrative and cleaning duties, which enhance overall service quality. However, further work is required to define optimum downtime in this domain.

A major strength of this study was its prospective design and real-time data capture. Although clerical, anaesthetic, nursing and porter staff were unaware of the study, radiologists and radiographers had insight into the study. The latter may have contributed to enhanced efficiency in certain aspects of the workflow.

This study is broadly applicable to global MR practice, in the public and private sectors, in well and poorly resourced environments, providing a new look at an old problem. It is hoped that this will serve as a benchmark and stimulate comparative studies in other institutions and healthcare systems.

## Conclusion

A critical analysis of MR ‘pre-scan’ and ‘post-scan’ times can provide valuable insights into opportunities for enhanced service efficiency.
